# Respect for illiterate or unconscious patient’s autonomy as a requirement for the legality of medical procedures in the polish healthcare system: a case report and review of the literature

**DOI:** 10.1186/s13037-022-00337-6

**Published:** 2022-08-31

**Authors:** K. Kocańda, S. Głuszek, M. K. Szerla, M. Domagała

**Affiliations:** Collegium Medicum, Jan Kochanowski University of Kielce, Kielce, Poland

**Keywords:** Patient’s objection, Patient’s consent, Advance healthcare directive, Illiterate patient

## Abstract

According to the Polish law, each patient has the right to refuse to consent to a medical procedure, even if the refusal concerns a life-saving procedure. It may be difficult for a physician to accept this kind of decision. In each case, however, medical intervention requires patient’s consent. Its lack makes physician’s actions illegal. Such a situation becomes more complicated when the patient who is intellectually incompetent, unconscious or illiterate is unable to express a consent to a medical procedure. Then, the possibility and the need to document and prove the patient’s consent becomes crucial from the point of view of the legality of medical personnel’s conduct. In this article, two representative clinical cases are discussed in the context of the legal assessment of the physician’s conduct in the event of legal complications related to the process of consenting to medical treatment. The authors analyze ethical dilemmas and legal risks that doctors may face in the process of consenting to risky medical procedures by unconscious and illiterate patient.

## Legal and medical circumstances of providing health services at increased risk

Progress in medical science has always been a source of enthusiasm and hope among patients and physicians alike, causing many ethical and legal doubts and reflections, at the same time. The current medical paradigm places a patient in the centre of interest in these fields of science, granting him or her respect and autonomy understood as competences regarding individual making important decisions about one’s own health and life.

A physician may perform surgery or apply a method of treatment or diagnosis posing an increased risk for a patient, after obtaining his or her written consent [1]. The Polish law does not define either surgery or any other medical procedure at an increased risk. The subject-matter literature states that the term “surgery”, as understood today, was firstly used as early as in 1597. Currently, in the medical term, surgery is defined as a medical act consisting in performing a maneuver or a series of maneuvers with or without the use of surgical instruments in order to treat a disease [2]. The term “increased-risk procedure” used in medical terminology is also not defined by the Polish legislator in any way. The only regulation specifying the need to obtain patient’s consent because of an increased-risk procedure only applies to patients admitted to a psychiatric hospital without their consent, and concerns a cisternal or lumbar puncture for collecting cerebrospinal fluid or administering drugs and performing electroconvulsive treatment [3].

According to the PWN Dictionary of Polish Language [4], “increased” means higher than normal, more intense. In a colloquial and fairly common understanding, surgery is a medical procedure performed in an operating theatre by a physician specialising in surgery on a patient being anaesthetised by an anaesthesiologist. Such a procedure often, but not always, involves tissue disruption. Therefore, it should be assumed that the category of an increased-risk medical procedure is a concept broader than surgery, often performed outside an operating theatre, for example, in endoscopic, invasive cardiology, and invasive radiology laboratories. A high-risk procedure is a dynamic and individual category, because it is a derivative of the health of a patient undergoing surgery, current medical knowledge, necessary skills and competences of both a surgeon and an anaesthesiologist, as well as appropriate resources (equipment, drugs) needed to perform a given procedure.

Medicine has made tremendous progress over the last 30 years. Possibilities of performing complicated and, at the same time, less invasive procedures have significantly expanded, thanks to the wide use of technologically advanced devices. The availability of technologically advanced devices supporting many organs and systems of patients is a milestone in the development of anaesthesiology and intensive care medicine. The human body’s response to surgical manipulations is proportional to the extent and duration of surgery. The effects of interference in a human body also include a post-surgery period. Therefore, the anaesthetic procedure includes elements of intra- and post-surgery intensive care. The development of new surgical techniques using modern devices provides surgical teams with the possibility of selecting a treatment method appropriate for an individual patient. The development of medical knowledge and skills as well as the possibility of using modern devices have significantly minimised risks connected with surgery. Thanks to the progress in this field, complicated surgical procedures have become possible even for patients at the borderline age, for example, infants and premature infants, and elderly persons, including patients whose clinical condition is burdened with not only with current diseases, but also coexisting chronic diseases, including those limiting patients’ intellectual competences.

At the beginning of the 1990s, the medical community adopted the concept of perioperative medicine understood not as a separate medical specialisation, but as a special care for each patient from the moment of making a decision about surgery, through the surgical period, to full recovery at home. This concept primarily takes into account the set of clinical symptoms and the pathophysiology of vital systems and organs important for humans’ life, which have or may have an impact on the clinical course of a patient qualified for surgery in the pre-, intra- and post-surgery period. The bases for optimising patient’s perioperative safety are: thorough assessment of patient’s health in terms of potential risk; patient, surgeon and anaesthesiologist’s joint decision making; selection of such medical procedure technique which minimises a perioperative risk and shortens a post-surgery period [5].

A dynamic nature of procedures categorised as those at an increased risk means that a treatment process should also take into account the knowledge about the type and severity of potential complications, the possibility of preventing them and/or reversing their negative effects. The individual nature of an increased risk is closely related to the specific health conditions of a patient which may substantially affect the nature and extent of the risk associated with a specific procedure. For these reasons, it seems reasonable to assume that a higher-risk procedure should be understood as any medical procedure that carries a greater risk for an individual patient than the existing risk for the majority of patients undergoing the same procedure.

Performing surgery or a medical treatment at an increased risk for a patient who is minor, incapacitated or incapable of expressing consent in writing, requires a substitute consent. Substitute consent in the case of incapacitated children or patients, i.e. legally equated in legal status to minors, is expressed by their statutory representative, who is usually a parent in the case of children, and a guardian or probation officer appointed by the court when it comes to incapacitated patients. In a situation where a patient is of legal age and incapacitated, or when it is impossible to communicate with the legal representative of a minor or incapacitated person, performing surgery or a medical treatment at an increased risk is possible after obtaining a consent made by the guardianship court [6].

In medical practice, however, there are situations where the fulfilment of this stringent legal condition is significantly difficult or even impossible, because a patient is in a state of immediate threat to his or her life and therefore he or she requires emergency surgery. If a delay caused by a consent-obtaining procedure would pose a threat to patient’s life, serious injury or serious health impairment, a physician may perform such medical procedures without the substitute consent of the patient’s statutory representative or the consent of the competent guardianship court. In such a case, the physician is obliged, if possible, to ask another physician, possibly of the same medical specialisation, for advice. Then, after carrying out all necessary treatment activities for the patient, the physician is obliged to immediately notify his or her statutory representative, actual guardian or the guardianship court about the performed activities [7].

## Advance healthcare directive in case of unconsciousness

In 2005, the Polish Supreme Court issued a judgement which became an important guideline in the legal assessment of patients’ declarations of will expressed in the event of loss of consciousness. There is no system of common law in Poland, but the judgements of the Supreme Court play the role of quasi precedents in practice due to the judiciary authority of this court [8]. The factual circumstances, the judgement issued was based on, concern a road accident the claimant suffered on 18 August 2004. As a result of it, she lost consciousness, and her health condition due to serious injuries, required transfusions of blood and blood products. A written declaration of will found with the patient, entitled “Health statement - no blood”, stipulated that she would not agree “under all circumstances” to “any form of blood transfusion”, even if it would be necessary to save her health and life. The woman also explained in her statement that she was one of Jehovah’s Witnesses and that she wanted to obey the Bible’s commandments, among which there was one telling: “Abstain from … blood” [9]. As a result of the accident participated by the woman, her husband died and her son also suffered.

The physicians, despite the fact that they knew the contents of the patient’s statement, applied to the guardianship court for permission to perform a blood transfusion. A court of first instance issued this kind of consent, pointing to the overriding need in the system of social values to save human life, which justified subjecting the claimant to medical procedures specified by a specialist in anaesthesiology and intensive care medicine as necessary. Based on the consent granted by the court, the patient underwent the transfusion of blood and its products, contrary to the instruction contained in her written health statement. After full recovery, she appealed against the court’s decision, challenging its lawfulness. At the stage of appeal proceedings, the claimant’s son joined the case as a participant. A court of second instance supported the arguments of the district court and discontinued the proceedings, but the claimant appealed against this decision to the Supreme Court.

When examining a cassation appeal filed in that case, the Supreme Court indicated that the regulations being in force in Poland regarding patient’s consent or its lack, despite the legal climate conducive to respecting patient’s will, do not apply directly to advanced directives, although similar regulations already exist in many countries (Patiententestament, testament de vie, living will). At the same time, the Supreme Court noted that there was no provision which would exclude patient’s right to determine which medical procedures should be abandoned in the event of patient’s loss of consciousness and his or her inability to effectively object to them. Therefore, it considered this type of declaration of the patient’s will to be assessed in this case as legally permissible and binding by deciding in the thesis of the judgement that the patient’s statement in the event of loss of consciousness, defining the will regarding physician’s conduct in relation to the patient in therapeutic situations that may arise, is for the physician – if it has been made explicitly and unambiguously - binding.

In the Convention for the Protection of Human Rights and Dignity of the Human Being with regard to the Application of Biology and Medicine of Oviedo, signed by Poland but still not ratified by it [10], it is indicated that the previously expressed wishes of the person concerned regarding the medical intervention should be taken into account, if at the time of its implementation he or she is not able to express his or her will. As indicated in the subject-matter literature, this regulation is categorical and constitutes an imperative of the need to respect such a declaration of will, which, however, does not apply to this type of patient’s wish being against the law, medical knowledge or physician’s conscience [11]. It may be treated as an interpretative guideline when assessing such cases under the Polish law. It expresses the axiom of respecting patient’s will regardless of the fact that his or her declaration of will is not expressed at the time of performing or resigning from a medical procedure, but in a way for the future, updating itself in connection with the fact that due to, for example, loss of consciousness, the patient cannot effectively express this will at a given moment.

The current standards of medical practice, which constitute Evidence Based Medicine (EBM), assume that the declaration of a Jehovah’s Witness, an adult and fully legally capable person, must be respected, and physicians should be prepared to use alternative methods of medical treatment. Patient’s decision, in physicians’ opinion may be wrong, but it must be respected by them [12]. Thus, despite the lack of an explicit legal regulation, patient’s will expressed for the future in the event of loss of consciousness, regardless of religious, ideological or other motives, is considered by judicature, legal doctrine and EBM as binding for physicians and requires its absolute respect.

### Presentation of a representative clinical case

It is a common situation in hospitals when it is necessary to urgently perform a higher-risk procedure or surgery, however, a patient is not able to sign a consent document due to certain physical obstacles, although he or she is fully conscious. Such a case took place during the hospitalisation of an over seventy-year-old patient from one of the hospitals in Poland [13], who expressed verbal objection to a blood transfusion procedure, but due to his health condition (bilateral paresis of the upper limbs), he could not sign the relevant document by hand. The patient was admitted to the hospital with a femur fracture. A diagnosis indicated: contact difficult due to deafness, correct mental orientation. The patient was unable to write by hand, but he was fully aware and acted with due discernment. On 8 December 2019, the patient, in the presence of his family, gave his consent orally to the surgical treatment of a femur fracture, which also includes blood transfusion, indicated in a therapeutic instruction. On 10 December 2019, a fracture anastomosis was performed, which resulted in blood loss, as a result of which the patient required a blood transfusion. On 11 December 2019, he orally refused the blood transfusion for the first time. The extensive written objection, drawn up by the physician receiving this declaration of will, did not bear the patient’s handwritten signature, but was articulated to the physician orally in the presence of the patient’s family. On 13 December 2019, the patient orally refused the blood transfusion again. The refusal to consent to the blood transfusion was expressed by the patient two times more, each time being recorded in a written form without the patient’s signature, but in the presence of witnesses. The family was insisting on the hospital performing the blood transfusion for the patient who suffered atherothrombotic stroke on 17 December 2019 against the patient’s will being known to the physicians.

Pursuant to the provision of Art. 79 of the Polish Civil Code [14], a person unable to write may submit a written declaration of will in such a way that he or she will make an ink fingerprint on a document, and a person authorised by him or her will write his or her name and surname and sign it next to this print, or in such a way that, instead of the person making the declaration, a person authorised by him or her will sign, and his or her signature will be certified by a notary public, a mayor of a municipality, a governor of a county, or a marshal of a province, with an indication that it was put on the document at the request of the person unable to write. Under the Polish law, a person who is unable to write, who has the ability to discern the essence of activities performed, may express his or her will either through an ink fingerprint in the presence of witnesses, or by using the official formula of expressing consent. It may seem that expressing the will by an illiterate person by making an ink fingerprint on a document requires the possibility of moving his or her hand, because performing this action must be an independent act, and the opposite interpretation could be a source of abuse. By analogy, it may be assumed that since a patient is not able to sign himself or herself, he or she is also not able to make an ink fingerprint on a document himself or herself. Involving an official, on the other hand, in a decision-making process, before whom patient’s will for a specific medical procedure may be expressed, seems difficult to enforce in practice, and it will not be applicable in emergency situations, when the need to obtain consent is so urgent that it is not possible to organise such a type of activity in the required time.

Such a situation took place in the described factual circumstances, because the patient due to bilateral paresis of his upper limbs was not able to sign the document of consent and then objection to a medical procedure. Because of the urgent nature of the medical activity, it was also not possible to use the official formula for signing a declaration of will by an illiterate person. In such cases, it seems that the only option is to receive patient’s declaration of will and document it in all possible ways which will sufficiently prove his or her will. It is possible to record patient’s statements, under his or her oral consent, by using sound or video recording devices. It should be assumed that the issue of the method of documenting a consent or objection to a medical procedure in a situation where a written form is objectively impossible is of enormous evidential significance, as it determines whether a physician can prove that he or she has performed a given medical procedure legally.

## Discussion

Human safety was and still is one of the highest values in almost all cultures and civilisations. Lack of safety causes anxiety and a sense of threat in both a child and an adult. Abraham Maslow, an outstanding American psychologist of the twentieth century, who also graduated in law, included safety among the elementary needs of every human being. Many fields of science deal with the study of safety aspects, including philosophy, psychology, medicine, and law. The interdisciplinary approach makes it possible to notice the multidimensionality of person’s safety, pointing to ethical and deontological standards for ensuring medical safety, as well as legal norms recognising safety as a protected value [15].

Medical risk is an inseparable element accompanying every patient undergoing the process of diagnosis and treatment. This is a danger that cannot be completely eliminated, but must and can be minimised. In each health care system, the safety of a person treated is the resultant of several basic factors: human, technical and environmental ones - and depends on the dynamic process of managing the risks associated with these factors throughout the course of treatment. For optimal patient’s safety, it is important to be aware of risk factors, as it allows them to be identified and eliminated in advance, avoiding the emergence of a risky situation crisis. Optimisation of patients’ safety is based primarily on compliance with procedures covering the organisation of medical activities, including careful qualification and preparation of patients. There are tools for this, such as guidelines and standards established by the bodies of scientific societies of all medical specialisations based on the best evidence medical research (BEMR). It is extremely important to identify patients at increased and high risk resulting from the coexistence of chronic systemic diseases. Such identification allows physicians to optimise patient’s condition before surgery, individualise diagnostic and treatment procedures, choose a type and method of surgery and anaesthesia. In the immediate post-surgery period, it allows for considering the patient’s treatment as intensive care or in an intensive care room [16].

It should be noted that limiting ourselves to algorithms as procedures based on BEMR may pose a specific risk of underestimating the variability of individual reactions of a human organism under the influence of context factors which define the individual situation of a specific person. Contextualisation is a process of identifying specific factors in patient’s life situation, focused on personalised care. In the light of the subject-matter literature, contextualisation is an integral part of therapy participated actively by a patient and/or his or her caregivers. Among many factors which make up the functioning of a person, family and socio-material situation, access to professional health care, and the possibility of exercising self-care are the main contextual factors directly and indirectly influencing patient’s health condition. The benefit of contextualisation in the treatment of an individual patient is still not sufficiently widespread, although it is an important element of a decision-making process, with a proven impact on the effectiveness and quality of care and patient’s satisfaction [17]. The implementation of each medical procedure for a patient should take into account the reduction of risks associated with it. Therefore, it is worth emphasising at this point that additional and equally important tools, enhancing patient’s safety, are also provided by broadly understood medical law, integrated in its content with medical determinants. Starting from identifying personal information through informed consent and ending with exercising due diligence in carrying out medical procedures.

For the legal validity of the statement of an adult and incapacitated patient made in the event of loss of consciousness, it is of prime importance to establish the authenticity of the signature on the document containing the content of his or her statement. The physician does not have any professional qualifications to assess whether the signature belongs to a particular patient. It may be able to compare it with the signature previously placed in the medical records, but this is not the case for patients who have not been hospitalised at all in a given hospital or in the absence of access to data on previous patient’s stays. A handwritten signature may be illegible and cause serious doubts as to evidence which has to be resolved definitively at a given moment under pain of illegal performance of a medical procedure. It has a negative impact on the possibility of uninterrupted provision of health services. It is also possible that the content of a statement itself is so ambiguous that it would be difficult for a physician to determine the actual will of a patient, and as a result, he or she may not be able to establish what the patient did not wish for at the time the statement was made, not only with sufficient certainty, but even with high probability. There may also be additional, legally irrelevant, but not indifferent ethically or emotionally, arguments of people related to a patient, especially when they constitute a source of information about a potential change of decision, which the patient simply did not manage to reveal earlier.

The Polish legal system does not provide for the possibility of making medical decisions for an adult and incapacitated patient by members of his or her family. In a situation where a patient with full civil rights, who has reached 18 years of age, cannot express his or her will to a medical procedure himself or herself, it is necessary to obtain a substitute consent from a guardianship court. The Polish legislator does not provide for the possibility of an alternative solution. The substitute consent of the guardianship court is the only legal option in this type of cases, as long as patient’s condition allows for waiting for its issuance. In a situation where a delay related to the procedure of obtaining the substitute consent would risk serious health consequences for the patient, a physician may even perform a high-risk medical procedure without this consent and notify the guardianship court of this fact after that.

The possibility of making decisions for a patient by another person applies only to those who are minor and fully or partially incapacitated. A person who is over 13 years old may be completely incapacitated if, due to a mental illness, mental retardation or other type of mental disorder, especially alcohol or drug addiction, he or she is unable to guide his or her conduct. For an incapacitated person, custody is completely established, unless he or she is still under parental authority. An adult person may be incapacitated partially due to a mental illness, mental retardation or other mental disorder, especially alcohol or drug addiction, if the person’s condition does not justify total incapacitation, but the help is needed to conduct his or her affairs. A guardianship is established for a partially incapacitated person [18].

In theory, it is possible for a patient to grant a power of attorney in the event of loss of consciousness, in which he or she would give a family member or other person the power to make a decision for him or her in a situation when he or she is unable to do so himself or herself. In practice, numerous and serious doubts regarding this type of legal transaction should be indicated, including the lack of subjective and objective guarantees of protection of the patient against the actions of his or her attorney [19]. Equally problematic seems to be empowering a substitute decision maker to make medical decisions in the part concerning a decision-making process itself, because if a patient agrees or opposes a specific medical procedure, a decision-making process takes place in the sphere of his or her will. This cannot be carried out by the substitute decision maker, as such a maker assesses the necessity of performing a medical procedure based on his or her opinion in relation to the risks and benefits associated with carrying the medical procedure or not, previously presented to him or her [20]. Meanwhile, it is a patient who should evaluate the proposed medical procedure from the point of view of possible complications and consequences of its failure, and it is not possible to transfer this element of a decision-making process to another person in this respect. Only in a situation where the power of attorney concerns a specific medical procedure, for which a patient had already received comprehensive therapeutic instruction and carried out an internal volitional process regarding this procedure, it may be assumed that the patient had relatively effectively empowered another person to make a proper decision for him or her. Also in such a case, a change in circumstances cannot be ruled out. It would in fact invalidate the authorisation granted, for example, in the case of a change of a physician for whom a consent was granted. A blank and general medical power of attorney, in turn, should be regarded as deprived of legal value of effectiveness due to the lack of the attribute of awareness and information regarding the activity to which a consent or objection is to be expressed.

In the judgement being discussed, the Supreme Court determines the need to respect patient’s will, but in the Polish legal system a quasi-precedent, formally binding only in the case on which it was issued, should not constitute the basis for decisions made by physicians in emergency circumstances. The legislator is expected to have legal certainty, the knowledge of which will be sufficient to make a legal decision regarding patient’s health or life, while such statements raise serious doubts among representatives of legal science, thus being completely ambiguous for physicians [21].

In the case of each medical procedure, patient’s consent is preceded by a therapeutic instruction under which a physician is obliged to provide the patient or his or her statutory representative with accessible information about his or her health, diagnosis, proposed and possible diagnostic and treatment methods, foreseeable consequences of their use or not using them, treatment results and prognosis for recovery. In a situation when a patient makes an advance healthcare directive, even if it concerns an objection to a specific medical procedure that is yet to be performed, he or she is not able to anticipate the consequences of failure to do so. For these reasons, such a patient’s decision is incomplete as made in circumstances other than those at the moment when the necessity to use it is updated [22]. It is possible that in a situation of imminent threat to life, in the absence of a medical alternative, a patient would change his or her mind. However, when at the moment of updating the necessity to use an advance healthcare directive, he or she is unable to revise his or her opinion, for example, due to loss of consciousness, his or her potential - previously expressed - objection becomes questionable. In the circumstances of the case of the patient injured in the road accident, her statement was categorical and authentic, and it was confirmed in a later court trial; however, it is difficult to deny the supposition that, due to the death of her husband in the same accident, she might want to change the previously made and expressed in writing decision about a possible objection at that moment, which became real in completely different circumstances.

In Poland, there is no legal regulation for advance healthcare directives, although such regulations already exist in many countries, including Belgium, the UK, the Netherlands, Spain, Austria, Finland, and Hungary [23]. The Polish legal doctrine points to the negative effects of the lack of statutory solutions regarding the admissibility and rules for submitting declarations of will for the future by patients, considering their legal admissibility in the *numerus clausus* system of unilateral legal actions, among which they are not mentioned [24]. Meanwhile, due to the legislator’s lack of actions, it is a physician who is forced to make a legal assessment of declarations of will made by patients in the event of loss of consciousness with consequences for their life or health, but each time there is no guarantee that the decision made by him or her to grant or refuse such a document is proper.

In the case of an illiterate patient, the legislator also does not provide physicians with sufficient help, because the legal solutions which are generally provided as a way of expressing will by people unable to write date from 1964 and have been in force almost unchanged from that time until today, and thus do not fit in with the present reality in any way. These regulations may be applied only partially, in the event that there are real, temporary and organisational possibilities to provide a patient with the possibility of submitting his or her declaration of will before an official such as a notary public. Involving such authorities as municipality mayors, county governors or province marshals seems completely redundant and practically impossible, given the political dimension of these positions and the scale of the potential demand for participation in activities with patients. Current medical knowledge which physicians are obliged to apply is dynamic, while the almost half-century-old legal solution concerning the way of documenting wills by patients unable to write is completely inconsistent with the challenges of social standards of dealing with patients.

Regardless of the lack of a contemporary feature, the existing legal regulation is incomplete as it does not regulate cases where, due to dynamic changes in patient’s health condition, it is not possible to call an official to document his or her will. In such situations, it seems necessary to use all the legally available options to record patient’s decision to consent or oppose a medical service, even by using audio-visual devices under the patient’s consent. However, if there is no consent to record patient’s oral statement, it is necessary to arrange the participation of witnesses in such activities, who shall then give a guarantee of credibility for a statement made by the patient. Obviously, physicians’ choices are limited to a group of people present at a hospital ward or simply available at a given moment. It is also be crucial to prepare a document that faithfully reflects the real will of a patient, but at the same time, contains all the obligatory elements for a physician, in particular, an accessible instruction of the patient about the consequences of expressed objection or the consequences of consent or declared objection. It seems that despite the lack of proper regulations at the level of generally applicable legislation, some internal instructions of a medical entity may be helpful in indicating the method of conduct of a physician in the event of inability to apply an imperfect legal solution, in order to minimise the need to independently search for ad hoc substitute solutions in case of emergency.

## Conclusions

According to the Polish Criminal Code, anyone who performs a medical procedure without patient’s consent is subject to a fine, restriction of liberty or imprisonment for up to 2 years [25]. Determining patient’s will in a situation where he or she previously expressed a potential objection in writing, i.e. articulated in the conditions of ignorance about the medical consequences of such objection, or when he or she cannot sign an objection or consent document at the time of expressing them, should be made with due diligence and with available methods and means.

A physician is obliged to practice in accordance with the indications of current medical knowledge, methods and ways of preventing, diagnosing and treating diseases available to him or her, according to the principles of professional ethics and with due diligence [26]. In the same way, a physician is obliged to seek to know and document the actual will of a patient and should then be assessed through this prism, taking into account the need to replace ad hoc the legislator who has refrained from sanctioning a specific model of conduct in such situations.

The physician’s obligation is an obligation of careful action, not the result, and by analogy to the nature of the obligation that is imposed on the physician in terms of the manner of performing his or her profession, it is appropriate to evaluate his or her conduct in the process of determining and documenting the content of patient’s actual will in a situation where he or she encounters the difficulties discussed in this regard.

Therefore, physician’s conduct should be assessed by the judicial authorities, which should confront his or her diligence in establishing and consolidating patient’s declaration of will about consent or objection to a medical procedure with the legislator’s carelessness in meeting the requirement of certainty and up-to-date legal provisions. Summarising the above considerations in the aspect of performing medical activities, the law should be a tool to optimise the safety of both people being treated (patients) and those who treat (medical personnel).

## Data Availability

Please contact the author for data requests.
